# Aphid fecundity and defenses in wheat exposed to a combination of heat and drought stress

**DOI:** 10.1093/jxb/eraa017

**Published:** 2020-01-14

**Authors:** Haicui Xie, Jianqin Shi, Fengyu Shi, Haiyun Xu, Kanglai He, Zhenying Wang

**Affiliations:** 1 College of Agronomy and Biotechnology, Hebei Normal University of Science and Technology, Qinhuangdao City, Hebei Province, China; 2 College of Life Science, Hebei University, Baoding City, Hebei Province, China; 3 State Key Laboratory for Biology of Plant Diseases and Insect Pests, Institute of Plant Protection, Chinese Academy of Agricultural Sciences, Beijing, China; 4 University of Birmingham, UK

**Keywords:** Drought, heat, phytohormones, resistance, *Sitobion avenae*, *Triticum aestivum* L

## Abstract

Plants are routinely subjected simultaneously to different abiotic and biotic stresses, such as heat, drought, and insect infestation. Plant–insect interactions in such complex stress situations are poorly understood. We evaluated the performance of the grain aphid (*Sitobion avenae*) in wheat (*Triticum aestivum* L.) exposed to a combination of heat and drought stresses. We also performed assays of the relative water content, nutritional quality, and responses of phytohormone signaling pathways. Lower relative water content and accumulation of soluble sugars and amino acids were observed in plants exposed to combined heat and drought stress. These conditions increased abscisic acid levels in the absence of aphids, as well as leading to higher levels of jasmonate-dependent transcripts. The grain aphid infestation further increased abscisic acid levels and the abundance of jasmonic acid- and salicylic acid-dependent defenses under the combined stress conditions. Aphids reared on plants grown under drought stress alone showed lower net reproductive rates, intrinsic rates of increase, and finite rates of increase compared with aphids reared on plants in the absence of stress. The heat-treated plants also showed a decreased aphid net reproductive rate. These findings demonstrate that exposure to a combination of stresses enhances plant defense responses against aphids as well as altering nutritional quality.

## Teaser

Combined heat and drought stress can enhance phytohormone-dependent defenses of wheat against aphids and decrease aphid fitness, providing new insights on plant–insect interactions under conditions of complex stress.

## Introduction

In recent years, climate change and extreme weather events have become a key focus of increasing attention worldwide (Intergovernmental Panel on Climate Change, 2018). The frequency of heatwaves has increased in most parts of the world, and the resulting instability in precipitation has led to increases in the total arid surface area at high and intermediate latitudes (Li *et al.*, 2014). Among the various abiotic stresses, heat and drought are two critical threats to crop growth and sustainable agriculture worldwide (Zhou *et al.*, 2017). In addition, herbivores are the key biotic stress affecting crop growth and yields in agroecosystems (Deutsch *et al.*, 2018). For these reasons, clarifying the expected changes in plant–insect interactions under combined heat and drought stress is an important objective in order to reduce insect damage to crops.

Reductions in the activity of various photosynthetic enzymes and water use efficiency can inhibit photosynthesis in many plant species under combined heat and drought conditions, which alters the primary metabolism of plants and thus disturbs the balance of carbon and nitrogen in plant tissues (Zhao *et al.*, 2013; Awasthi *et al.*, 2014; Li *et al.*, 2014; Elferjani and Soolanayakanahally, 2018; Zandalinas *et al.*, 2018). For example, previous studies have indicated that combined heat and drought cause an accumulation of sucrose that potentially protects mitochondria and other cellular components from the adverse effects of drought (Rizhsky *et al.*, 2004). These combined stresses also increase the accumulation of free amino acids, which are partially converted into proline to relieve abiotic stress (Cvikrova *et al.*, 2013; Feller and Vaseva, 2014). At the same time, plant responses to a variety of adverse stresses can activate phytohormone signaling pathways (Lee and Luan, 2012; Danquah *et al.*, 2014). Typically, the abscisic acid (ABA) signaling pathway, which also regulates the jasmonic acid (JA) and salicylic acid (SA) signaling pathways, can be stimulated by heat and drought stress in plants; these signaling pathways are interconnected via a complex network (Adie *et al.*, 2007; Asselbergh *et al.*, 2007, 2008; Zandalinas *et al.*, 2018). Furthermore, up-regulation of the SA and JA signaling pathways are two key plant defense responses induced by insect feeding (Koornneef and Pieterse, 2008). Thus, the combined effects of heat and drought might affect carbon and/or nitrogen in host plants through effects on the primary metabolism and the resistance of plants to herbivores (McDowell, 2011).

Insects can also be significantly influenced by environmental factors, including heat and drought (Huberty and Denno, 2004; Colinet *et al.*, 2015). The growth and development of insects has been shown to be directly and indirectly influenced by heat. For example, the magnitude and frequency of heatwaves can directly affect the metabolic rate, developmental duration, survival rate, and adult fecundity of insects (Neven, 2000; Ma *et al.*, 2015). Moreover, the chemical composition of host plant tissues often changes under heat, which indirectly affects the feeding and fitness of insects in the context of specific host plants (Jamieson *et al.*, 2015, 2017). Many insects are indirectly affected by changes in host plants grown under drought conditions, owing to reductions in water content and changes in the contents of sugars, some amino acids, and secondary metabolites (Huberty and Denno, 2004). However, the response of a particular insect species can vary among different plants grown under drought stress (Mewis *et al.*, 2012). 

Until now, the effects of combined heat and drought on plant–insect interactions have remained unclear. One of the few relevant studies indicated that the survival of *Lochmaea suturalis* (Thomson) was strongly and mostly negatively influenced by three commonly considered climate change factors—drought, warming, and elevated CO_2_ (Scherber *et al.*, 2013). The additive effects of temperature and drought stress on *Melanaspis tenebricosa* (Comstock) led to increased female embryo production and body size with increasing temperature, such that these response variables were greater in insects reared on drought-stressed trees compared with sufficiently watered trees (Dale and Frank, 2017). Thus, more work is need to explain the intrinsic mechanisms underlying changes in plant–insect interactions under heat and drought stress.

The grain aphid, *Sitobion avenae*, is one of the most dominant and destructive pests of wheat worldwide (Blackman and Eastop, 2000). Crop nutritional quality and defense responses are important factors in aphid feeding, growth, and population size. When wheat is fed on by aphids, plant defenses are coordinated by several interacting signaling systems, especially the JA and SA signaling pathways (Felton and Korth, 2000; Ding *et al.*, 2016), which appear to be regulated by the ABA signaling pathway (Guo *et al.*, 2016; Nachappa *et al.*, 2016). For aphids feeding on wheat, soluble sugars and free amino acids are important sources of carbon and nitrogen (Caputo and Barneix, 1999; Chen *et al.*, 2004). 

The current study had the following specific goals: (i) to quantify the effects of heat and drought on relative water content, nutritional quality, and phytohormone-dependent defenses in wheat, and (ii) to determine the performance of aphid populations reared on wheat under heat and drought conditions. The results of our study will inform pest control strategies in the context of global climate change, and perhaps suggest that aphids and other sap-feeding pests will become less of a problem for wheat cultivation.

## Materials and methods

### Plant preparation and temperature and drought treatments

The experiment was conducted in six environmental chambers, of which three chambers were maintained at ambient temperature (22 °C under 16 h of light and 18 °C under 8 h of dark) and three chambers were maintained at an elevated temperature (22 °C under 12 h of light, 30 °C under 4 h of light, and 18^o^C under 8 h of dark). The environmental chambers were maintained at 60–70% relative humidity (RH). Plants were grown in 9.0 cm deep plastic pots (7.5 cm diameter) filled with a sterilized loamy field soil (organic carbon content 75 g kg^–1^) in each environmental chamber. Plants were irrigated with tap water, and pot weights were measured twice per day to maintain 20% soil water content (SWC) for the well-watered treatment and 10% SWC for the drought stress treatment. The heat and drought stress were imposed simultaneously when the wheat seedlings emerged in each environmental chamber. Each pot was planted with one seedling, and 30 pots were used for each water level in each environmental chamber. Plants from 15 pots were used for the aphid feeding experiment, and the remaining 15 pots were used for testing indicators in wheat. 

For phytohormone analysis, when plants were at the two-leaf stage, 20 wingless *S. avenae* adults were transferred to the first leaf (i.e. the oldest leaf) of each plant. After feeding for 24 h, all aphids were removed, and leaf samples were then collected, immediately frozen in liquid nitrogen, and kept at –80 °C until analysis (Zhang *et al.*, 2017). Uninfested leaves were used as controls and treated as described above. The experiment was performed in three consecutive replicates. All environmental chambers were used for each treatment in different replicates.

### Plant relative water content

The fresh weight (FW) of the leaves was measured and the leaves were then rehydrated in distilled water for 24 h at 15 °C in darkness and weighed again to obtain their weight at full turgor (TW). The leaves were then dried to a constant weight to obtain estimates of their dry weight (DW). Leaf relative water content (RWC) was calculated using the formula RWC (%)=(FW−DW)×100/(TW−DW) (Barrs and Weatherley, 1962).

### Measurement of phytohormones, soluble sugars, and amino acids in plants

To assay amino acid, soluble sugar, and phytohormone contents, an HPLC-MS/MS system was used, which was composed of a Shimadzu UHPLC system (Nexera UHPLC LC-30A; Shimadzu Corp., Kyoto, Japan) and an AB Sciex QTRAP^®^ 6500 mass spectrometer (AB Sciex, Redwood City, CA, USA) equipped with an autosampler, ESI electrospray ionization source, and TSQ mass analyzer.

To quantify the amino acids and soluble sugar concentrations in phloem, phloem exudates were obtained from three leaves per plant using the EDTA exudation technique described by Douglas (1993) and Tetyuk *et al.* (2013). Phloem exudates were immediately frozen in liquid nitrogen and kept at –80 °C.

For amino acid analysis, the phloem exudates and 35 ml of pure water were transferred into 50 ml volumetric flasks and shaken. Then, 500 μl of the solution was transferred into a test tube and mixed with 250 μl phenyl isothiocyanate–acetonitrile (1.2%, v/v) and 250 μl triethylamine–acetonitrile (4%, v/v). After incubation for 1 h at room temperature. 50 μl of acetic acid (20%, v/v) was added, and the lower layer of the solution was used as the sample. Aliquots of 10 µl of each sample were injected into the HPLC-MS/MS system. Amino acids were separated with an ACQUITY BEH C18 column (100 mm × 2.1 mm, 1.7 μm; Waters Corporation, Milford, MA, USA) under gradient conditions, using 5 mM ammonium acetate (A) and acetonitrile (B) as the mobile phases, at a flow rate of 0.3 ml min^–1^. The gradient program for quantification of amino acids is shown in Supplementary Table S1 at *JXB* online. The column was maintained at 30 °C.

For soluble sugar analysis, phloem exudates and 35 ml of 40% acetonitrile were transferred into 50 ml volumetric flasks, followed by ultrasound extraction for 30 min and dilution with 40% acetonitrile to 50 ml for use as samples. Then, 10 µl aliquots of each sample were injected into the HPLC-MS/MS system. Sugars were separated with a Waters BEH Amide column (4.6 mm × 250 mm, 5 µm; Waters Corporation), using 0.1% triethylamine (A) and acetonitrile containing 0.1% triethylamine (B) as the mobile phases, with isocratic elution at a flow rate of 1.0 ml min^–1^. The column was maintained at 30 °C.

For phytohormone analysis, samples of frozen leaves (0.2 g) were homogenized in liquid nitrogen. The resulting homogenate and 10 ml of ethyl acetate were transferred into a 25 ml centrifuge tube, followed by ultrasound extraction for 20 min and centrifugation for 10 min at 21 130 *g*. The supernatant was evaporated to dryness under a stream of nitrogen at 40 ℃, and the final extracts were dissolved in 1 ml of 70% methanol and used as samples for analysis. Then, 10 µl aliquots of the samples were injected into the HPLC-MS/MS system. Phytohormones were separated with an Acquity UPLC® BEH C18 column (2.1 mm × 100 mm 1.7 µm; Waters Corporation) under gradient conditions, using 0.1% formic acid (A) and methanol (B) as the mobile phases, at a flow rate of 0.3 ml min^–1^. The gradient program for phytohormone quantification is shown in Supplementary Table S2. The column was maintained at 30 °C.

### Determination of JA- and SA-related gene expression by quantitative RT–PCR

A Quick-RNA™ MiniPrep Kit (TR154-50; Zymo Research Corporation, Irvine, CA, USA) was used to isolate total RNA from leaf samples. The quality and quantity of the RNA were assessed with a NanoReady FC3100 spectrophotometer (FC-1100). A 1 μg aliquot of RNA was reverse transcribed into cDNA with 5× All-In-One RT MasterMix (with AccuRT Genomic DNA Removal Kit; Applied Biological Materials, Vancouver, Canada), and cDNA templates were stored at –20 °C until they were used for quantitative reverse transcription PCR (RT–qPCR). Target genes for the JA-responsive pathway included *lipoxygenase* (*LOX*) and *allene oxide synthase* (*AOS*), which are involved in JA biosynthesis (Liu *et al.*, 2011). The genes assessed for the SA-responsive pathway were the SA synthesis enzymes *phenylalanine ammonia lyase* (*PAL*) and the induced SA marker protein *pathogenesis-related protein 1* (*PR-1*) (Chen *et al.*, 2009). Actin was used as an internal control and was amplified using the primer sequences described by Liu *et al.* (2011). Specific primers for genes were designed from *T. aestivum* expressed sequence tag sequences using Primer Premier 5.0 (Zhang *et al.*, 2017). All primer sequences are listed in Supplementary Table S3 (Zhang *et al.*, 2017).

RT–qPCR was performed on an ABI Q6 Flex Real-Time PCR System (Applied Biosystems, Foster City, CA, USA). The PCRs were performed in 20 μl reaction volumes containing 1 μl of cDNA, 0.4 μl each of 10 μmol l^–1^ forward and reverse primers, 10 μl of 2× Taq Master Mix, and 8.2 μl of double distilled H_2_O, under the following thermal cycling conditions: 2 min at 95 °C followed by 40 cycles of 10 s at 95 °C and 34 s at 60 °C, with a final 10 min at 72 °C.

### Aphid life history parameters

A single *S. avenae* clone was collected from a wheat field in Langfang City, Hebei Province, China, and its progeny was subsequently reared on wheat plants (‘Beijing 837’) for 5 years in an environmental chamber at 20±1 °C, 60–70% RH, and a 16 h:8 h light:dark photoperiod. Wheat plants in different treatments were used to rear *S. avenae* in the environmental chambers described above. One newly emerged nymph (<6 h old) was used to infest the abaxial side of a wheat leaf. The plants were then confined in transparent plastic column cages (6.4 cm diameter × 20.0 cm in height) covered with nylon netting on the top to prevent the aphids from escaping. Molting, survival, and the number of offspring were recorded daily until all parental aphids had died. The progeny were recorded and removed daily. Three experimental replicates were conducted for each treatment, which consisted of 45 individually caged aphids.

### Statistical analyses

All data were analyzed using the statistics package SAS version 9.2 (SAS Institute, Cary, NC, USA). For RT–qPCR, the fold changes in the expression of target genes were calculated using the 2^–△△Ct^ normalization method (Livak and Schmittgen, 2001). Age-specific reproduction of aphids was used to construct a life table (Birch, 1948). For the life table parameters, intrinsic rate of increase (*r* m) was computed using the Euler equation: $\sum_{x=0}^{\infty }e^{rx}l_{x}m_{x}=1$, where *l*_*x*_ is survivorship of the original cohort over the age interval from day *x*–1 to day *x* (i.e. pivotal age) and *m*_*x*_ is the mean number of female offspring produced per surviving female during the age interval *x*. The other parameters examined, including net reproductive rate (*R*_0_), generation time (*T*), and finite rate of increase (λ), were calculated as described by Maia *et al.* (2000). The effects of heat, drought, and aphid infestation on ABA, JA, and SA content and on JA- and SA-related gene expression in plants were tested by three-way ANOVAs. The effects of heat and drought on plant relative water content, soluble sugar content, and free amino acid content in plant phloem and on aphid life table parameters were tested by two-way ANOVAs. Least significant difference tests were used to determine whether treatment means differed significantly when ANOVAs indicated that a factor was significant. For all analyses, *P*<0.05 was considered as the threshold for statistical significance. All data were checked for normality and equality of residual error variances, and were appropriately transformed if necessary to satisfy the assumptions of ANOVA.

## Results

### Relative water content changes in wheat grown under heat and drought stress

**Fig. 1.**
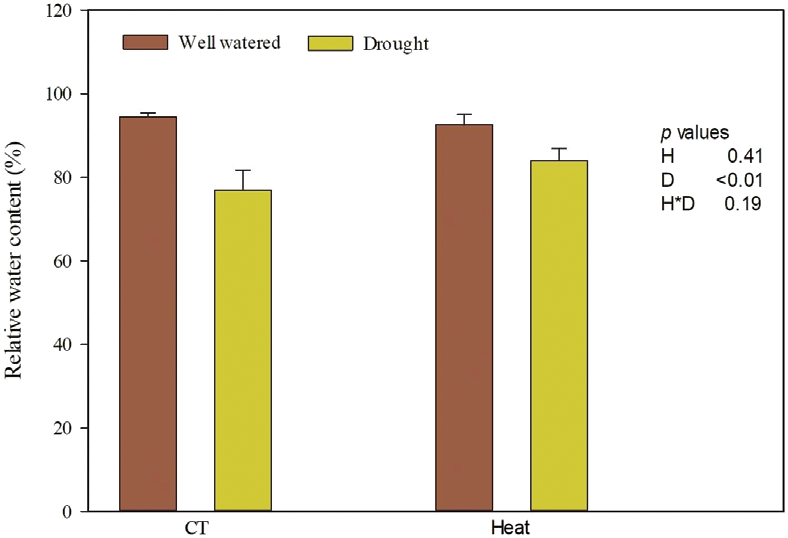
Relative water content of wheat grown under heat and drought conditions. Each value represents the mean ±SE of three replicates. *P* values are provided for two-way ANOVA on the effects of heat (H) and drought (D) treatments on relative water content. CT, control temperature. (This figure is available in colour at *JXB* online.)

Leaf relative water content showed differing changes in response to heat and drought (Fig. 1, Supplementary Table S4). Relative to leaves exposed to the well-watered treatment, the relative water content of leaves under drought stress was 13.91% lower (Fig. 1). However, heat stress did not influence leaf relative water content (Fig. 1). The interactions between heat stress and drought stress on the relative water content of wheat leaves were not significant (Supplementary Table S4).

### Changes in nutrient quality in wheat grown under heat and drought stress

Heat and drought stress significantly promoted amino acid accumulation in wheat (Table 1, Supplementary Table S5). Heat stress was associated with higher glutamate, tryptophan, proline, tyrosine, aspartic acid, asparagine, and total amino acid contents, but lower glycine content in wheat, compared with the control temperature treatment (Table 1, Supplementary Table S5). Drought stress was associated with higher phenylalanine, glutamate, proline, tyrosine, tryptophan, aspartic acid, asparagine, and total amino acid contents, compared with the well-watered treatment, (Table1, Supplementary Table S5). Furthermore, there were significant positive interaction effects between heat stress and drought stress on phenylalanine, valine, tyrosine, and aspartic acid contents (Supplementary Table S5), and significant negative interaction effects between heat and drought stress on alanine, glycine, arginine and threonine Supplementary Table S5).

**Table 1.** Amino acid contents (μg g^–1^) of wheat grown under heat and drought conditions (mean ±SE, *n*=3)

Treatment	Leucine	Phenylalanine	Alanine	Methionine	Glycine
Control temperature + well watered	22.18±3.33 Aa	14.82±2.29 B	129.64±12.99 Aa	2.89±0.20 Aa	10.62±0.54 a
Control temperature + drought	22.85±1.79 Aa	12.19±1.02 A	91.53±1.60 Aa	3.11±0.29 Aa	7.75±0.72 a
Heat + well watered	19.05±1.57 Aa	5.87±0.92 B	69.01±2.09 Aa	2.30±0.26 Aa	3.98±0.38 b
Heat + drought	28.42±1.57 Aa	16.57±1.58 A	141.22±15.89 Aa	2.82±0.15 Aa	4.50±0.50 b
	Glutamate	Glutamine	Valine	Arginine	Lysine
Control temperature + well watered	176.76±11.21 Bb	236.76±19.36 Aa	23.68±2.29 Aa	165.75±29.87 Aa	154.52±10.63 Aa
Control temperature + drought	218.25±21.39 Ab	218.25±21.39 Aa	39.32±2.17 Aa	160.19±13.54 Aa	180.23±23.49 Aa
Heat + well watered	225.31±12.53 Ba	225.31±12.53 Aa	39.32±2.17 Aa	146.22±14.0 Aa	154.168±18.33 Aa
Heat + drought	274.08±10.31 Aa	274.08±10.32 Aa	22.56±5.39 Aa	223.77±12.02 Aa	200.16±10.21 Aa
	Tyrosine	Proline	Tryptophan	Serine	Threonine
Control temperature + well watered	17.96±2.11 Bb	4.58±0.20 Bb	40.24±1.09 Bb	205.32±6.64 Aa	66.78±2.84 Aa
Control temperature + drought	21.167±0.68 Ab	12.35±1.47 Ab	75.33±3.68 Ab	204.17±7.80 Aa	33.29±1.34 Aa
Heat + well watered	18.49±1.52 Ba	8.53±0.72 Ba	45.62±1.76 Ba	188.94±10.25 Aa	44.11±3.57 Aa
Heat + drought	31.70±3.39 Aa	13.40±1.17 Aa	100.60±12.04 Aa	199.83±12.52 Aa	72.65±6.46 Aa
	Aspartic acid	Asparagine	Isoleucine	Serine	Total
Control temperature + well watered	30.46±1.14 Bb	1324.78±94.51 Bb	26.19±2.06 Aa	174.83±12.68 Aa	2828.76±114.90 Bb
Control temperature + drought	52.01±2.24 Ab	1825.09±221.19 Ab	32.26±7.24 Aa	172.58±2.74 Aa	3381.89±272.51 Ab
Heat + well watered	31.58±3.06 Ba	1917.97±236.51 Ba	32.67±1.60 Aa	132.764±10.17 Aa	3298.78±191.67 Ba
Heat + drought	69.59±5.85 Aa	2236.61±125.84 Aa	43.49±4.90 Aa	172.29±9.64 Aa	4128.35±184.86 Aa

Different lower-case letters indicate significant differences between the control temperature and heat treatments; different capital letters indicate significant differences between the well-watered and drought treatments as determined by a least significant difference test (*P*<0.05).

**Fig. 2.**
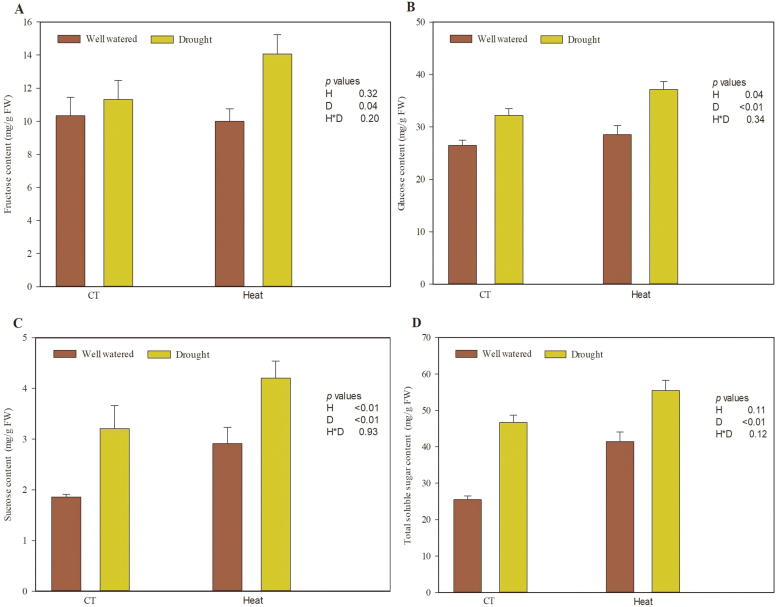
Soluble sugar content of wheat grown under heat and drought conditions: (A) fructose; (B) glucose; (C) sucrose; (D) total soluble sugars. Each value represents the mean ±SE of three replicates. *P* values are provided for two-way ANOVA on the effects of heat (H) and drought (D) treatments on relative water content. CT, control temperature. (This figure is available in colour at *JXB* online.)

Heat and drought stress significantly affected the soluble sugar content of plants (Fig. 2, Supplementary Table S6). Relative to wheat in the control temperature treatment, the glucose and sucrose concentrations were 12.0% and 26.9% higher, respectively, in wheat under heat stress (Fig. 2B, C). Relative to the well-watered treatment, drought stress increased fructose, glucose, sucrose, and total sugar contents, by 23.3%, 26.1%, 41.4%, and 23.4%, respectively (Fig. 2). The interaction effects between heat and drought stress on the soluble sugars in wheat were not significant (Supplementary Table S6).

### Phytohormone-dependent defense against aphids of wheat under heat and drought stress

**Fig. 3.**
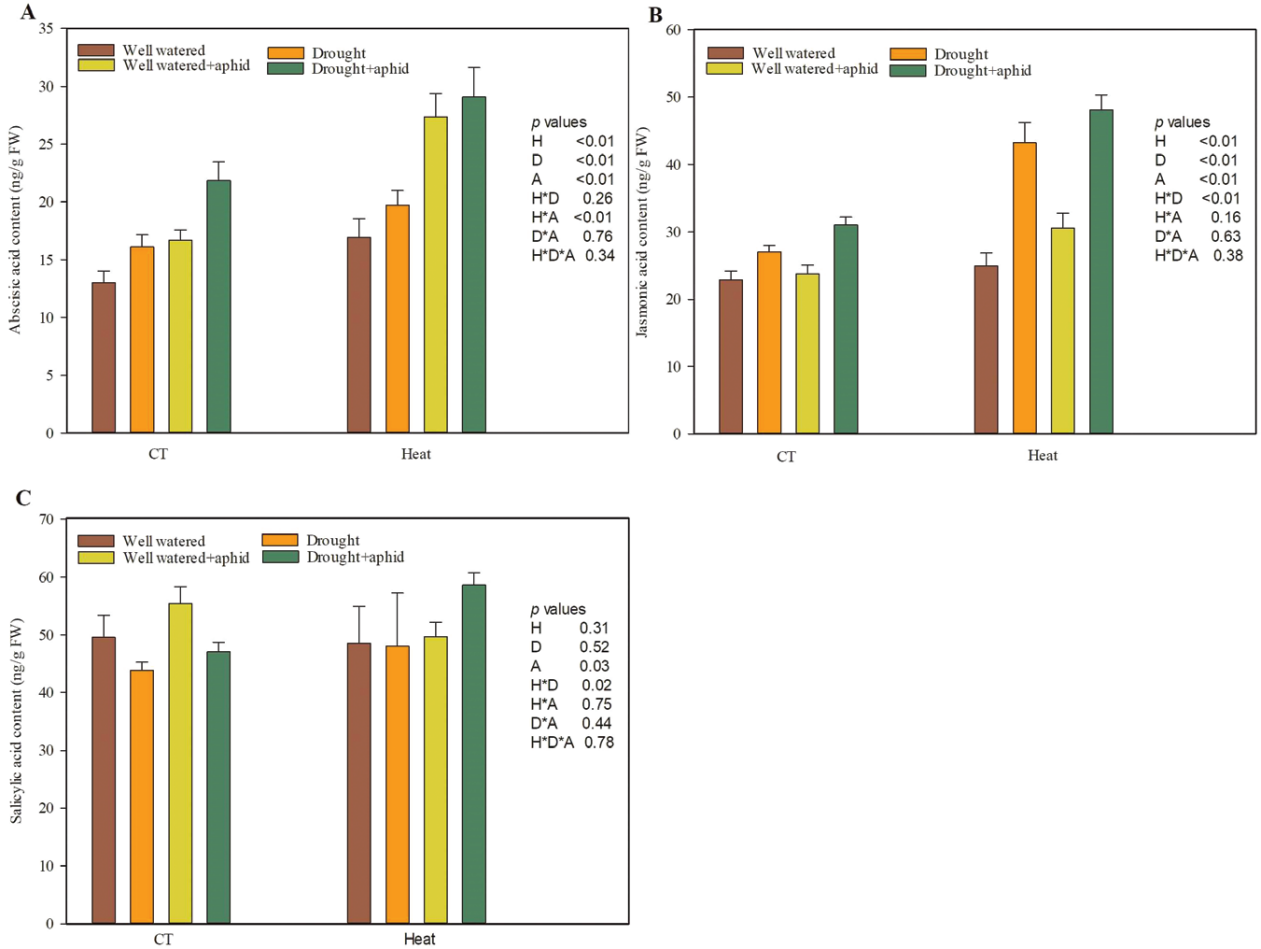
Phytohormone content of wheat grown under heat and drought conditions with and without grain aphid infestation. (A) Abscisic acid; (B) jasmonic acid; (C) salicylic acid. Each value represents the mean ±SE of three replicates. *P* values are provided for two-way ANOVA on the effects of heat (H), drought (D), and aphid (A) treatments on relative water content. CT, control temperature. (This figure is available in colour at *JXB* online.)

Heat, drought, and aphid infestation significantly increased the phytohormone content of wheat (Fig. 3, Supplementary Table S7). Both heat and drought stress significantly increased the ABA and JA contents in wheat, compared with wheat grown under the control temperature and well-watered treatments (Fig. 3A, B). Aphid infestation significantly increased the ABA, JA, and SA contents in wheat compared with uninfested plants (Fig. 3, Supplementary Table S7). Furthermore, there were significant positive interaction effects between heat and infestation on ABA, and between heat and drought on JA and SA (Supplementary Table S7).

**Fig. 4.**
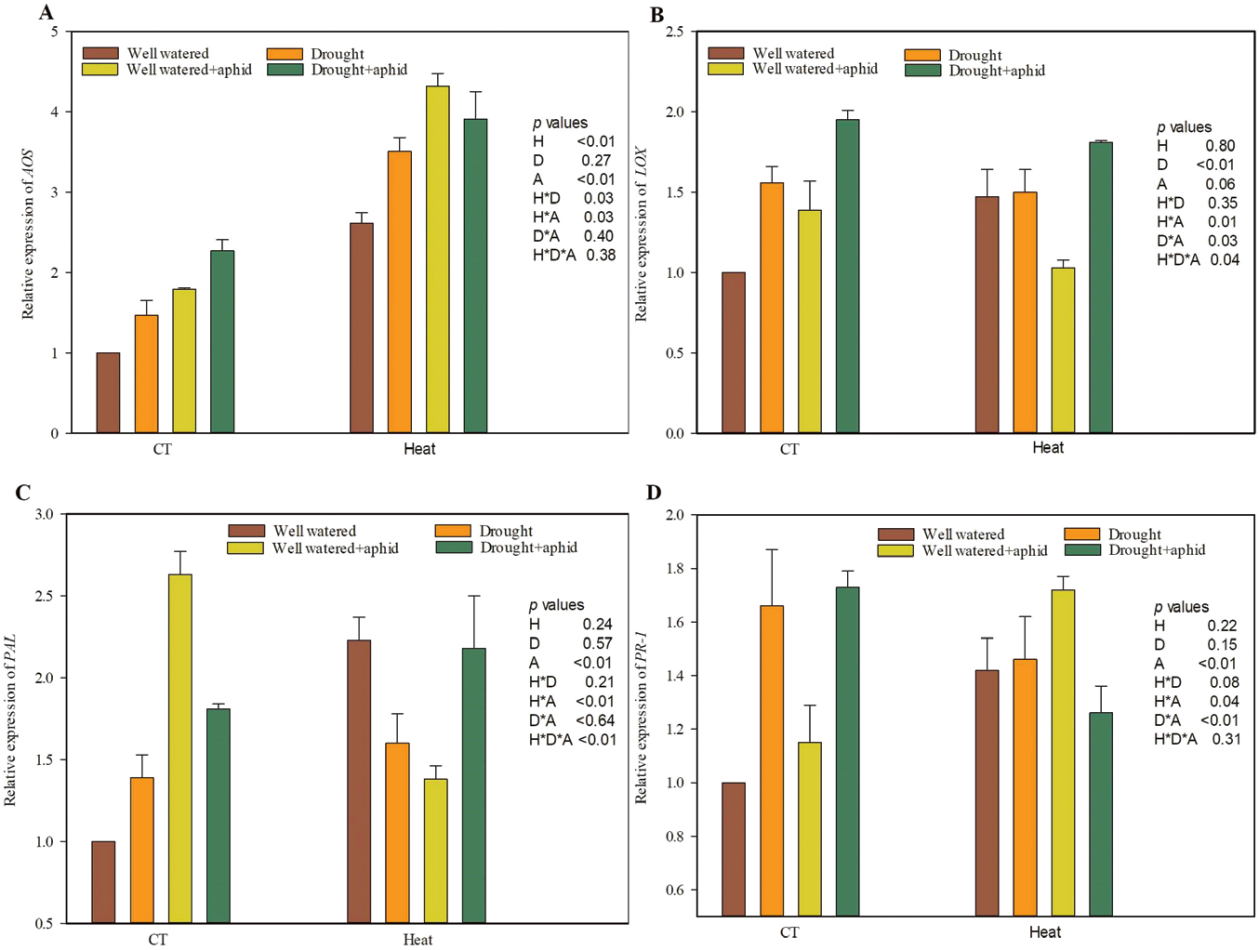
Jasmonic acid- and salicylic acid-related gene expression of wheat grown under heat and drought conditions with and without grain aphid infestation. (A) *AOS*; (B) *LOX*; (C) *PAL*; (D) *PR-1*. Each value represents the mean ±SE of three replicates. *P* values are provided for two-way ANOVA on the effects of heat (H), drought (D), and aphid (A) treatments on relative water content. CT, control temperature. (This figure is available in colour at *JXB* online.)

The relative expression of genes involved in the JA and SA defense signaling pathways were examined in wheat (Fig. 4, Supplementary Table S8). The expression of *AOS* was significantly up-regulated in wheat grown under heat stress compared with the control temperature treatment (Fig. 4A). The expression of *LOX* was significantly up-regulated in wheat grown under drought stress compared with the well-watered treatment (Fig. 4B). At the same time, aphid-infested wheat plants showed up-regulation of the expression of *AOS*, *PAL*, and *PR-1* compared with uninfested plants (Fig. 4A, C, D, Supplementary Table S8). Furthermore, there were significant positive interaction effects between heat and drought stresses on the expression of *AOS*; between heat and infestation on the expression of *AOS*, *PAL*, and *PR-1*; between drought stress and infestation on the expression of *LOX* and *PR-1*; and between heat, drought, and infestation stresses on the expression of *LOX*. There was also a significant negative interaction between heat and infestation on the expression of *LOX* (Supplementary Table S8).

### Changes in life table parameters for grain aphids feeding on wheat under heat and drought stress

**Fig. 5.**
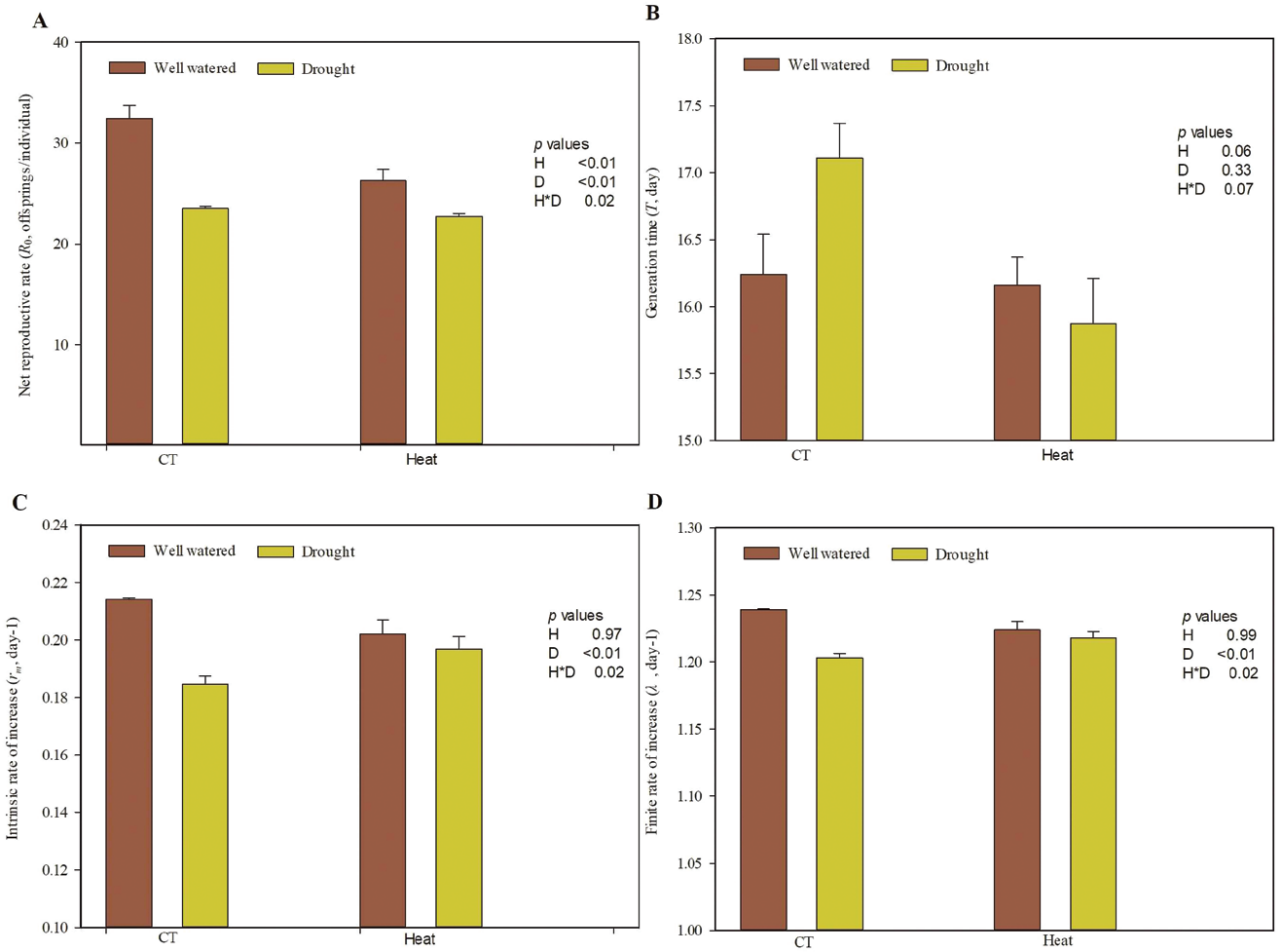
Life table parameters of aphids reared on wheat grown under heat and drought conditions. (A) Net reproduction rate (*R*_0_); (B) generation time (*T*); (C) intrinsic rate of increase (*r*_m_); (D) finite rate of increase (λ). Each value represents the mean ±SE of three replicates. *P* values are provided for two-way ANOVA on the effects of heat (H) and drought (D) treatments on relative water content. CT, control temperature. (This figure is available in colour at *JXB* online.)

Both heat stress and drought stress significantly influenced the life table parameters of grain aphids feeding on wheat (Fig. 5, Supplementary Table S9). The *R*_0_ values of aphid populations were lower under heat stress compared with the control temperature treatment (Fig. 5A). The *R*_0_, *r*_m_, and λ values of aphid populations were lower under drought stress compared with the well-watered treatment (Fig. 5A, C, D). Furthermore, there were significant positive interaction effects between heat and drought stresses on *R*_0_, *r*_m_, and λ (Supplementary Table S9).

## Discussion

### Drought decreased the relative water content of wheat

Our results indicated that drought significantly decreased the relative water content of wheat, but heat did not. As shown by Zhou *et al.* (2017), drought stress had a dominant effect over heat stress on plants subjected to combined stresses. The invariant plant water status under heat stress might be a consequence of the combined effects of changes in osmotic potential and intrinsic water use efficiency (Pérez-Romero *et al.*, 2019). In many cases, continuous drought stress resulted in a significant reduction in the foliar water content of host plants, which has a negative impact on sap feeder abundance (Huberty and Denno, 2004). That is, the lower water content may interfere with the ability of pests to access or utilize sources of nutrition (Huberty and Denno, 2004; Guo *et al.*, 2016).

### Heat and drought stress increased the nutritional quality of wheat

The accumulation of amino acids, sugars, and other substances is coordinated with the activation of specific physiological and molecular responses in plants grown under heat and drought stresses, which mitigate the damaging effects of combined stresses (Zandalinas *et al.*, 2018) but also provide more nutrition to insects (Chen *et al.*, 2004).

Accumulation of amino acids can also be associated with rapid metabolic recovery of plants after stress, which could thus also mitigate the damage caused by abiotic stress (Suguiyama *et al.*, 2014). In this study, both heat and drought stress significantly increased the accumulation of proline, glutamate, tyrosine, tryptophan, aspartic acid, asparagine, and total amino acids (Table 1). Similarly, numerous studies have shown that proline content increases under different environmental stresses (Szabados and Savouré, 2010). To enhance plant tolerance to drought and heat stresses, large quantities of proline are transported between the cytosol, chloroplasts, and mitochondria to complete other metabolic reactions in plant tissues (Feller and Vaseva, 2014; Kaur and Asthir, 2015). Tryptophan, tyrosine, and phenylalanine are aromatic amino acids that are located downstream of the shikimate pathway (Tzin and Galili, 2010). These amino acids increased in purslane under stress, suggesting a shift in metabolism toward secondary metabolite production, which indicates its important role in plant stress tolerance (Tzin and Galili, 2010). Thus, the accumulation of tryptophan, tyrosine, and phenylalanine might have promoted secondary metabolite production and JA-dependent defenses in wheat in this study. Glutamate is the common precursor of aspartic acid, asparagine, and other essential amino acids in plants (Azevedo *et al.*, 2006). Heat or drought has also been shown to increase the accumulation of glutamate in other plants (Good and Zaplachinski, 1994; Li *et al.*, 2016). Thus, the increased accumulation of aspartic acid and asparagine might be due to the increased accumulation of glutamate observed in this study. The accumulated amino acids are essential nutritional substrates for the aphids used in this study s(Douglas and Prosser, 1992; Sasaki and Ishikawa, 1995; Rabatel, 2013), except for proline, which may be acting as a stress-related signal more than as a nutrient substrate (Szabados and Savouré, 2010). We also found that there was a positive interaction between heat and drought stress in the accumulation of several amino acids, that is, the combination of heat and drought enhanced the accumulation of amino acids (Supplementary Table S5). Thus, the accumulation of amino acids enhances plant tolerance to drought and heat stress; however, this process might also regulate JA-dependent defenses and have an impact on the nutrients available to aphids feeding on wheat.

Previous studies have indicated that soluble sugars accumulated to higher levels in plants exposed to drought stress (Mohammadkhani and Heidari, 2008; Marček, 2019). In several plants subjected to combined heat and drought stress, sucrose accumulated, potentially to protect mitochondria and other cellular components from the adverse effects of abiotic stress (Rizhsky *et al.*, 2004; Jin *et al.*, 2015). Similarly, heat stress increased the glucose and sucrose contents in wheat (Fig. 2B, C). Drought stress increased the glucose, sucrose, fructose, and total sugar concentrations in wheat (Fig. 2). Thus, both heat and drought increased the accumulation of sugars in wheat. Additionally, the increased soluble sugar contents may have mitigated the adverse effects of heat and drought stress in wheat, while also increasing its nutrition quality to aphids.

### Heat and drought stress induced the up-regulation of phytohormone-dependent defense against aphids

The plant responses to the combination of abiotic stresses differed from those observed under individual stresses (Zandalinas *et al.*, 2018). Phytohormone signaling pathways have been reported to play critical roles in the response of plants to abiotic and biotic stresses; however, their role and regulation under combined stresses remain unclear (Gupta *et al.*, 2017). Plant responses to individual heat or drought stresses involve changes in the ABA, JA, and SA signaling pathways (Danquah *et al.*, 2014; Ahammed and Yu, 2016; Zandalinas *et al.*, 2016). Cross-talk between the ABA signaling pathway and other phytohormone signaling pathways, such as those of JA and SA, may modify the relationship between plants and herbivorous insects (Mauch-Mani and Mauch, 2005; Ding and Oldroyd, 2009; Fan *et al.*, 2009), as up-regulation of the JA and SA signaling pathways were two major insect-induced defense responses observed (Felton and Korth, 2000). Previous experiments indicated that ABA signaling pathway activity was positively correlated with JA signaling and negatively correlated with SA signaling pathway activity under abiotic stress (Mauch-Mani and Mauch, 2005; Ahammed and Yu, 2016; Guo *et al.*, 2016). Similarly, Ding *et al.* (2016) found that JA and SA showed antagonistic effects in pathogen-infected wheat. In agreement, our results indicated that ABA and JA content accumulated under both heat and drought stress. Furthermore, the expression levels of the JA defense-related genes *AOS* and *LOX* increased in wheat grown under heat stress and drought stress, respectively (Fig. 4). However, the SA content and the expression levels of the SA defense-related genes *PAL* and *PR-1* were unchanged in wheat grown under heat and drought stress. Thus, heat and drought stress increased ABA levels, which might have induced the up-regulation of JA-dependent defense, but did not influence SA-dependent defense in wheat in this study. The unchanged SA-dependent defense pathway might be due to inhibition by the ABA and JA signaling pathways and a significant interaction between heat and drought stress (Supplementary Table S7). The aphid infestation experiment showed that infestation significantly increased the levels of ABA, JA, and SA and the expression levels of JA and SA defense-related genes (i.e. *AOS*, *PAL*, and *PR-1*) even under heat and drought stress (Figs 3 and 4). Thus, the JA-dependent and aphid-induced defense responses against aphids were enhanced under heat and drought stress.

### Heat and drought stress directly and indirectly influenced the life table parameters for grain aphids feeding on wheat

The growth and development of insects can be directly influenced by external environmental factors and indirectly influenced by host resistance and nutritional quality (Clissold and Simpson, 2015). In this study, heat stress decreased the *R*_0_ of grain aphid populations; this observation could be explained by previous studies showing that heat stress had direct adverse effects on insect fecundity (Chiu *et al.*, 2015; Sales *et al.*, 2018). Moreover, the combination of heat and drought stress and aphid infestation enhanced the wheat defense response, but the nutritional quality of wheat was improved by heat and drought stress. At the same time, the lower relative water content induced by drought might also decrease the absorption of nutrition by aphids from wheat (Huberty and Denno, 2004). Overall, the direct heat stress, enhanced host defense response, and lower relative water content had obvious adverse effects on grain aphids, but the higher nutritional quality of wheat would have had beneficial effects on grain aphids under heat and drought stress. The combined effect of all the above characteristics was that the *R*_0_*, r*_m_, and λ values of aphid populations on wheat decreased under heat and drought stress (Fig. 5A, C, D), indicating that adverse effects play a more important role in grain aphid reproduction under stress than the higher nutritional quality of the wheat under these stresses. This might be a consequence of grain aphids needing to overcome a variety of adverse effects, including JA- and SA-dependent defenses, before their stylets can reach the phloem sap in order to access sugars and amino acids located beneath the epidermis and mesophyll (Smith and Boyko, 2007; Guo *et al.*, 2014).

Our data demonstrate that heat and drought stress changed the interaction between wheat plants and grain aphids, mainly through the enhanced defense response and lower relative water content of host plants, but possibly also as a direct effect of heat stress on grain aphids. Furthermore, plant–insect interactions may vary with plant and insect species. Moreover, insect fitness over multiple generations of a host plant might also change under heat and drought stress. Thus, more research is needed to elucidate the mechanisms of interactions between plants and insects under heat and drought stress, which may help to predict the insect damage to crops in the context of projected climate change scenarios. This study suggests that, as heat and drought are becoming more frequent sources of stress to crops, fewer resources will need to be dedicated to remediating aphid infestations of wheat.

## Supplementary data

Supplementary data are available at *JXB* online.

Table S1. Gradient elution program in the HPLC analysis for amino acids.

Table S2. Gradient elution program in the HPLC analysis for phytohormones.

Table S3. RT–qPCR primers for genes involved in JA and SA defense responses.

Table S4. Summary of ANOVA results for effects of heat and drought on relative water content.

Table S5. Summary of ANOVA results for effects of heat and drought on amino acid contents.

Table S6. Summary of ANOVA results for effects of heat and drought on sugar contents.

Table S7. Summary of ANOVA results for effects of heat, drought, and aphid infestation on phytohormone contents.

Table S8. Summary of ANOVA results for effects of heat, drought, and aphid infestation on JA- and SA-related gene expression.

Table S9. Summary of ANOVA results for effects of heat and drought on aphid life table parameters.

eraa017_suppl_supplementary_tables_S1_S10_figure_S1
