# Supplementary material for: Aphid fecundity and defenses in wheat exposed to a combination of heat and drought stress
Source: J Exp Bot. 2020 Jan 14;71(9):2713–22. doi: 10.1093/jxb/eraa017 (PMC7210778; doi:10.1093/jxb/eraa017)
Supplement: eraa017_suppl_supplementary_tables_S1_S10_figure_S1 [file eraa017_suppl_supplementary_tables_s1_s10_figure_s1.pdf]

Table S1 Gradient elution program in the HPLC analysis for amino acids.

Time / min	A / %	B / %
0	95	5
1	95	5
1.5	80	20
4.5	80	20
5	50	50
6	50	50
6.5	0	100
7.5	0	100
8	95	5
12	95	5

Table S2 Gradient elution program in the HPLC analysis for phytohormone.

Time / min	A / %	B / %
1	75	25
1.5	35	65
3	35	65
3.5	75	25
6	-	-

Table S3. RT-qPCR primers for genes involved in JA and SA defense responses.

Primer	Primer sequences	Tm	PCR products	Temperature	E value
AOS-F	ACTTCAACACGCTCAACGACT	58	208bp	60°C	0.92
AOS-R	TCACCGCTGACAAAGATGG	57.81			
LOX-F	GACCAGCGAAACAACAACC	57.56	239bp		0.96
LOX-R	GCATACAATAGCGGGAACAC	57.8			
PAL-F	CCACCCTGGACAGATTGAA	57.56	214bp		0.98
PAL-R	ATGAGCGGGTTGTCGTTG	57.3			
PR-1-F	ATAACCTCGGCGTCTTCAT	55.41	164bp		1.01
PR-1-R	TACTCGCTCGGTCCCTCT	59.58			
Actin-F	GGAAAATCAGTCTCGGTTCAG	58.01	70bp		0.96
Actin-R	TCATACAGCAGGCAAGCAC	57.56			

Table S4. Summary of ANOVA results for effects of heat and drought on relative water content.

Treatment	*df*	*F*	*P*
Heat	1	0.75	0.41
Drought	1	17.59	< 0.01
Heat*drought	1	2.04	0.19

Table S5. Summary of ANOVA results for effects of heat and drought on amino acid contents.

Measurement	Treatment	*df*	*F*	*P*
Leucine	Heat	1	0.38	0.55
	Drought	1	5.33	0.06
	Heat*drought	1	4.01	0.08
Phenylalanine	Heat	1	2.21	0.18
	Drought	1	6.91	0.03
	Heat*drought	1	18.88	<0.01
Alanine	Heat	1	0.28	0.61
	Drought	1	2.72	0.14
	Heat*drought	1	28.42	<0.01
Methionine	Heat	1	3.66	0.09
	Drought	1	2.58	0.15
	Heat*drought	1	0.44	0.53
Glycine	Heat	1	81.13	<0.01
	Drought	1	4.57	0.07
	Heat*drought	1	9.56	0.02
Glutamate	Heat	1	12.87	<0.01
	Drought	1	9.62	0.02
	Heat*drought	1	0.06	0.81
Glutamine	Heat	1	1.8	0.22
	Drought	1	0.84	0.39
	Heat*drought	1	4.13	0.08
Valine	Heat	1	4.43	0.07
	Drought	1	3.09	0.12
	Heat*drought	1	9.64	0.02
Arginine	Heat	1	1.56	0.25
	Drought	1	4.16	0.08
	Heat*drought	1	5.56	0.05
Lysine	Heat	1	0.35	0.57
	Drought	1	4.65	0.06
	Heat*drought	1	0.37	0.56
Tyrosine	Heat	1	6.52	0.03
	Drought	1	14.37	0.01
	Heat*drought	1	5.43	0.05
Proline	Heat	1	6.14	0.04
	Drought	1	39.37	<0.01
	Heat*drought	1	2.06	0.19

Tryptophan	Heat	1	5.77	0.04
	Drought	1	49.85	< 0.01
	Heat*drought	1	2.43	0.16
Serine	Heat	1	1.17	0.31
	Drought	1	0.26	0.63
	Heat*drought	1	0.39	0.55
Threonine	Heat	1	4.34	0.07
	Drought	1	0.38	0.55
	Heat*drought	1	59.81	< 0.01
Aspartic acid	Heat	1	71.24	0.03
	Drought	1	7.02	< 0.01
	Heat*drought	1	5.44	0.05
Asparagine	Heat	1	7.79	0.02
	Drought	1	5.17	0.05
	Heat*drought	1	0.25	0.63
Isoleucine	Heat	1	3.77	0.09
	Drought	1	3.42	0.1
	Heat*drought	1	0.27	0.62
Serine	Heat	1	4.92	0.06
	Drought	1	3.81	0.09
	Heat*drought	1	4.79	0.06
Total	Heat	1	9.39	0.02
	Drought	1	12.07	< 0.01
	Heat*drought	1	0.48	0.51

Table S6. Summary of ANOVA results for effects of heat and drought on sugar contents.

Measurement	Treatment	*df*	*F*	*P*
Fructose	Heat	1	1.11	0.32
	Drought	1	5.77	0.04
	Heat *drought	1	1.96	0.20
Glucose	Heat	1	5.95	0.04
	Drought	1	24.7	< 0.01
	Heat *drought	1	1.01	0.34
Sucrose	Heat	1	9.75	0.01
	Drought	1	16.52	< 0.01
	Heat *drought	1	0.01	0.93
Total sugars	Heat	1	3.15	0.11
	Drought	1	15.25	< 0.01
	Heat *drought	1	3.01	0.12

Table S7. Summary of ANOVA results for effects of heat, drought, and aphid infestation on phytohormone contents.

Measurement	Treatment	*df*	*F*	*P*
	Heat	1	63.12	< 0.01

ABA	Drought	1	15.97	< 0.01
	Infestation	1	83.33	< 0.01
	Heat*drought	1	1.39	0.26
	Heat*infestation	1	10.38	< 0.01
	drought*infestation	1	0.10	0.76
	Heat*drought*infestation	1	0.98	0.34
JA	Heat	1	42.25	< 0.01
	Drought	1	79.53	< 0.01
	Infestation	1	19.95	< 0.01
	Heat*drought	1	45.08	< 0.01
	Heat*infestation	1	2.13	0.16
	drought*infestation	1	0.24	0.63
	Heat*drought*infestation	1	0.81	0.38
SA	Heat	1	1.11	0.31
	Drought	1	0.44	0.52
	Infestation	1	5.83	0.03
	Heat*drought	1	6.82	0.02
	Heat*infestation	1	0.11	0.75
	drought*infestation	1	0.64	0.44
	Heat*drought*infestation	1	1.98	0.18

Table S8. Summary of ANOVA results for effects of heat, drought, and aphid infestation on JA- and SA-related gene expression.

Measurement	Treatment	*df*	*F*	*P*
AOS	Heat	1	161.04	< 0.01
	Drought	1	1.28	0.27
	Infestation	1	67.35	< 0.01
	Heat*drought	1	5.32	0.03
	Heat*infestation	1	5.96	0.03
	drought*infestation	1	0.75	0.40
	Heat*drought*infestation	1	0.82	0.38
LOX	Heat	1	0.06	0.80
	Drought	1	53.29	< 0.01
	Infestation	1	4.1	0.06
	Heat*drought	1	0.95	0.35
	Heat*infestation	1	8.0	0.01
	drought*infestation	1	5.37	0.03
	Heat*drought*infestation	1	5.13	0.04
PAL	Heat	1	1.49	0.24
	Drought	1	0.34	0.57
	Infestation	1	15.88	< 0.01
	Heat*drought	1	1.77	0.21
	Heat*infestation	1	26.78	< 0.01
	drought*infestation	1	0.23	0.64

	Heat*drought*infestation	1	35.07	< 0.01
PR-1	Heat	1	1.65	0.22
	Drought	1	2.27	0.15
	Infestation	1	17.82	< 0.01
	Heat*drought	1	3.54	0.08
	Heat*infestation	1	5.21	0.04
	drought*infestation	1	23.56	< 0.01
	Heat*drought*infestation	1	1.11	0.31

Table S9. Summary of ANOVA results for effects of heat and drought on aphid life table parameters.

Measurement	Treatment	*df*	*F*	*P*
R_0	Heat	1	15.17	< 0.01
	Drought	1	48.38	< 0.01
	Heat*drought	1	8.97	0.02
T	Heat	1	5.47	0.06
	Drought	1	1.08	0.33
	Heat*drought	1	4.23	0.07
r_m	Heat	1	0.01	0.97
	Drought	1	18.57	< 0.01
	Heat*drought	1	9.01	0.02
λ	Heat	1	0.01	0.99
	Drought	1	18.47	< 0.01
	Heat*drought	1	8.95	0.02

Table S10. The mean CT values of actin in different treatments

Control temperature				Heat			
Water	Drought	Water+aphid	Drought+aphid	Water	Drought	Water+aphid	Drought+aphid
27.68	27.66	27.7	27.88	27.56	27	28.07	28.32

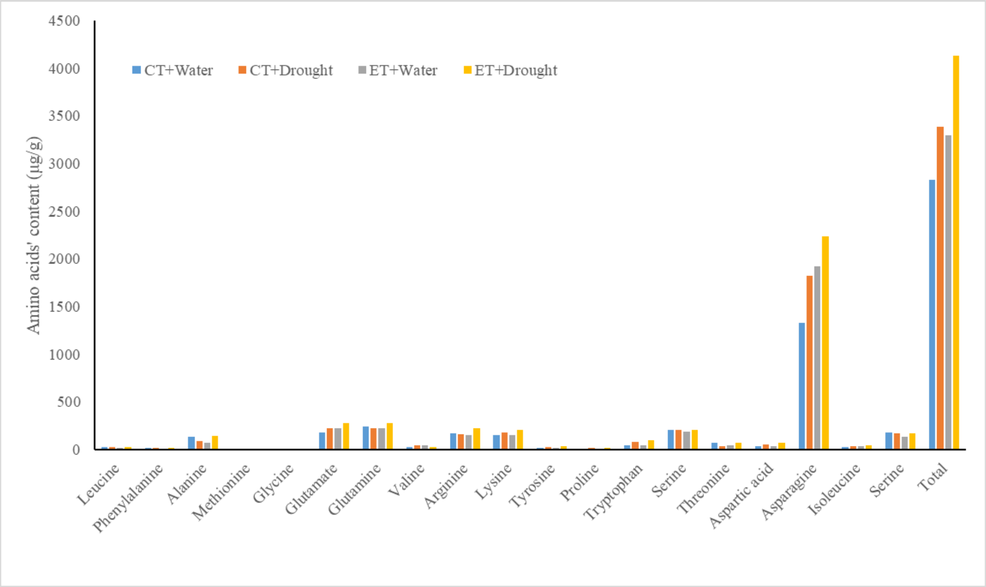

Fig. S1 Amino acid contents of wheat grown under heat and drought conditions
